# Supplementary figures and images for: Spatially resolved clonal copy number alterations in benign and malignant tissue
Source: Nature. 2022 Aug 10;608(7922):360–7. doi: 10.1038/s41586-022-05023-2 (PMC9365699; doi:10.1038/s41586-022-05023-2)

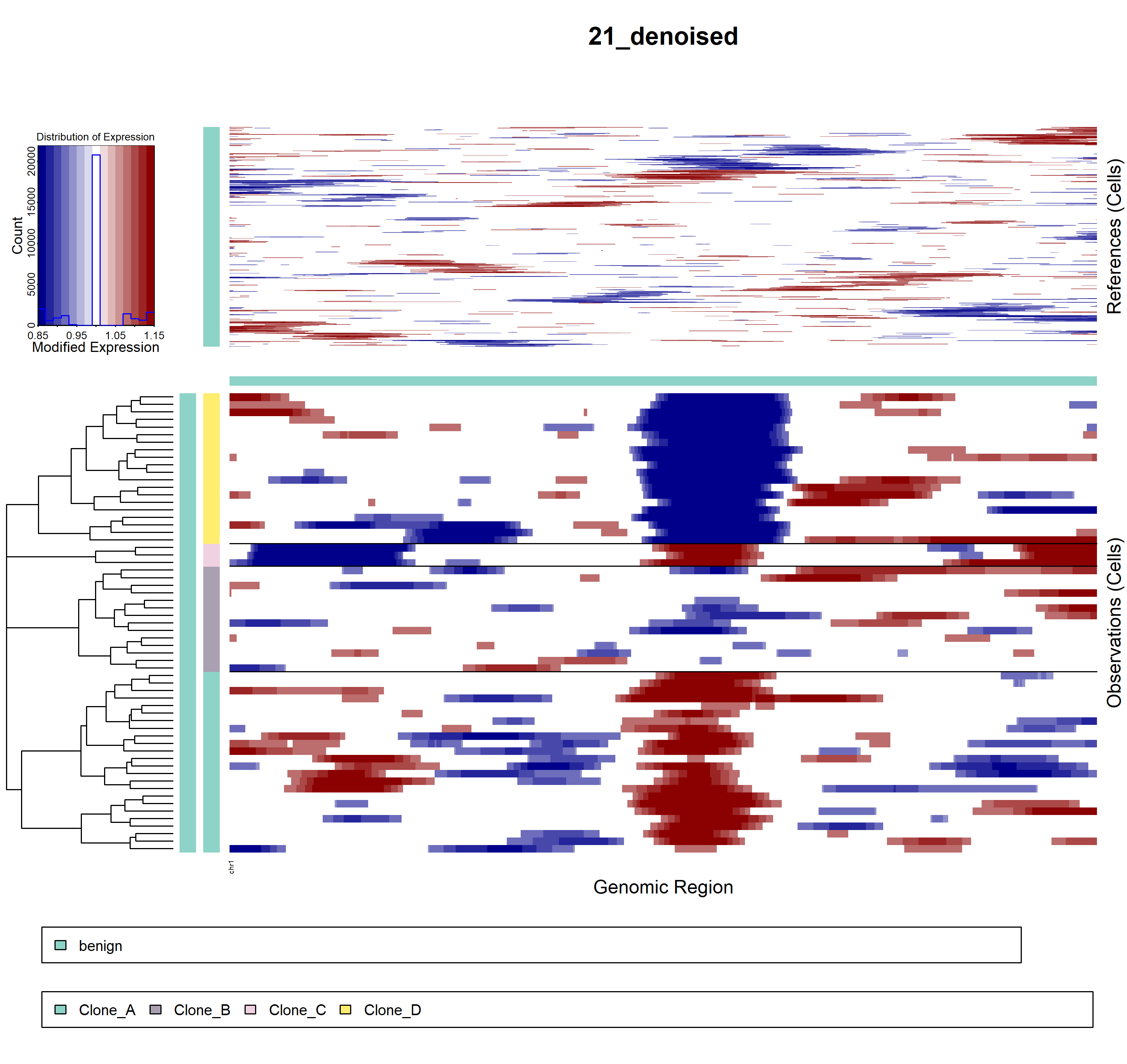

Supplement: Supplementary file 1 — Synthetic data generation and evaluation. Files related to method evaluation with synthetic data, such as the design file, the generated synthetic data, results from spatial inferCNV when applied to the synthetic data and a script for evaluation of results. [file 41586_2022_5023_MOESM1_ESM.zip › supplementary-data-S2/results/infercnv.21_denoised.png]

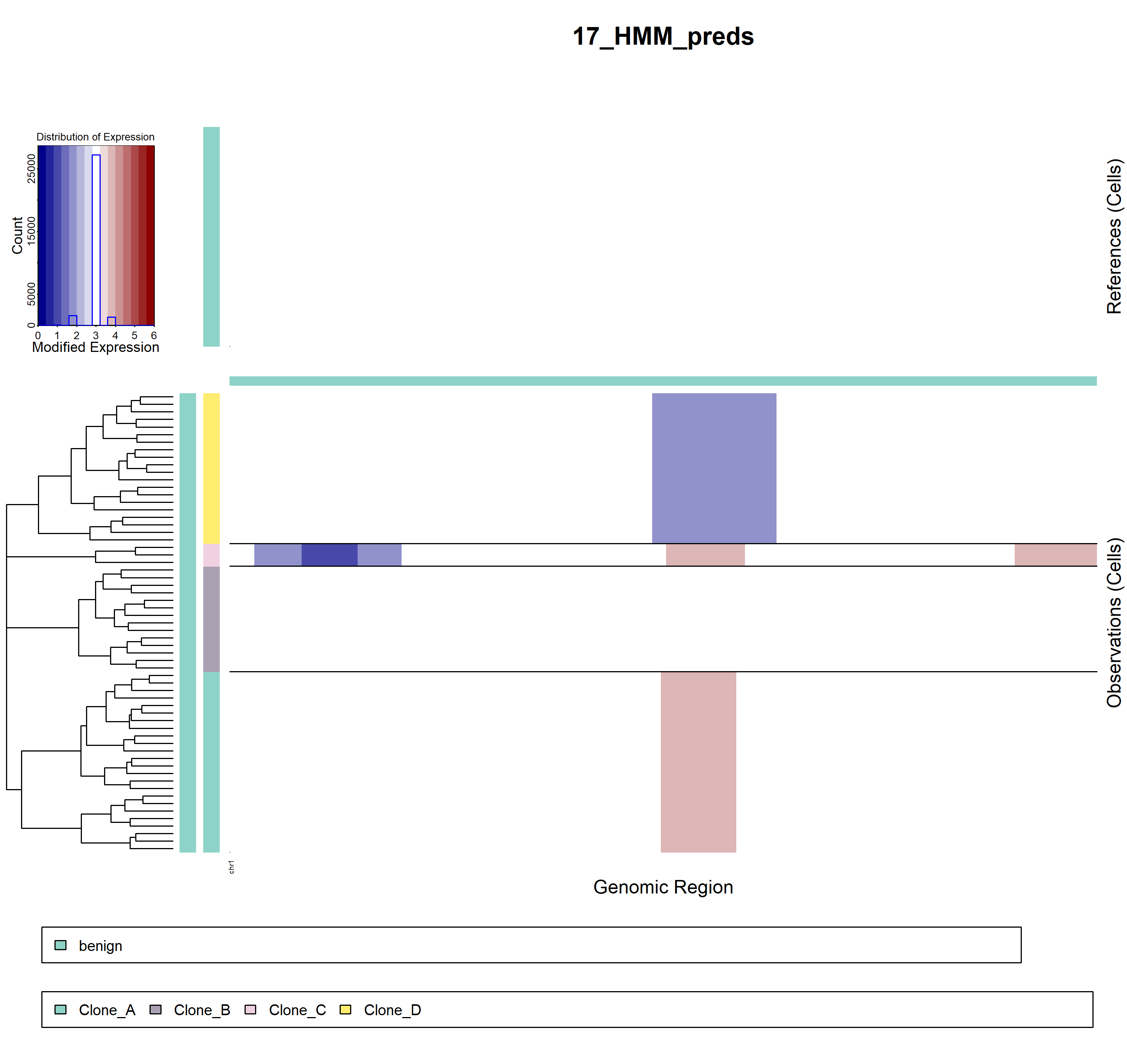

Supplement: Supplementary file 1 — Synthetic data generation and evaluation. Files related to method evaluation with synthetic data, such as the design file, the generated synthetic data, results from spatial inferCNV when applied to the synthetic data and a script for evaluation of results. [file 41586_2022_5023_MOESM1_ESM.zip › supplementary-data-S2/results/infercnv.17_HMM_predHMMi6.hmm_mode-samples.png]
