# Supplementary material for: Spatially resolved clonal copy number alterations in benign and malignant tissue
Source: Nature. 2022 Aug 10;608(7922):360–7. doi: 10.1038/s41586-022-05023-2 (PMC9365699; doi:10.1038/s41586-022-05023-2)

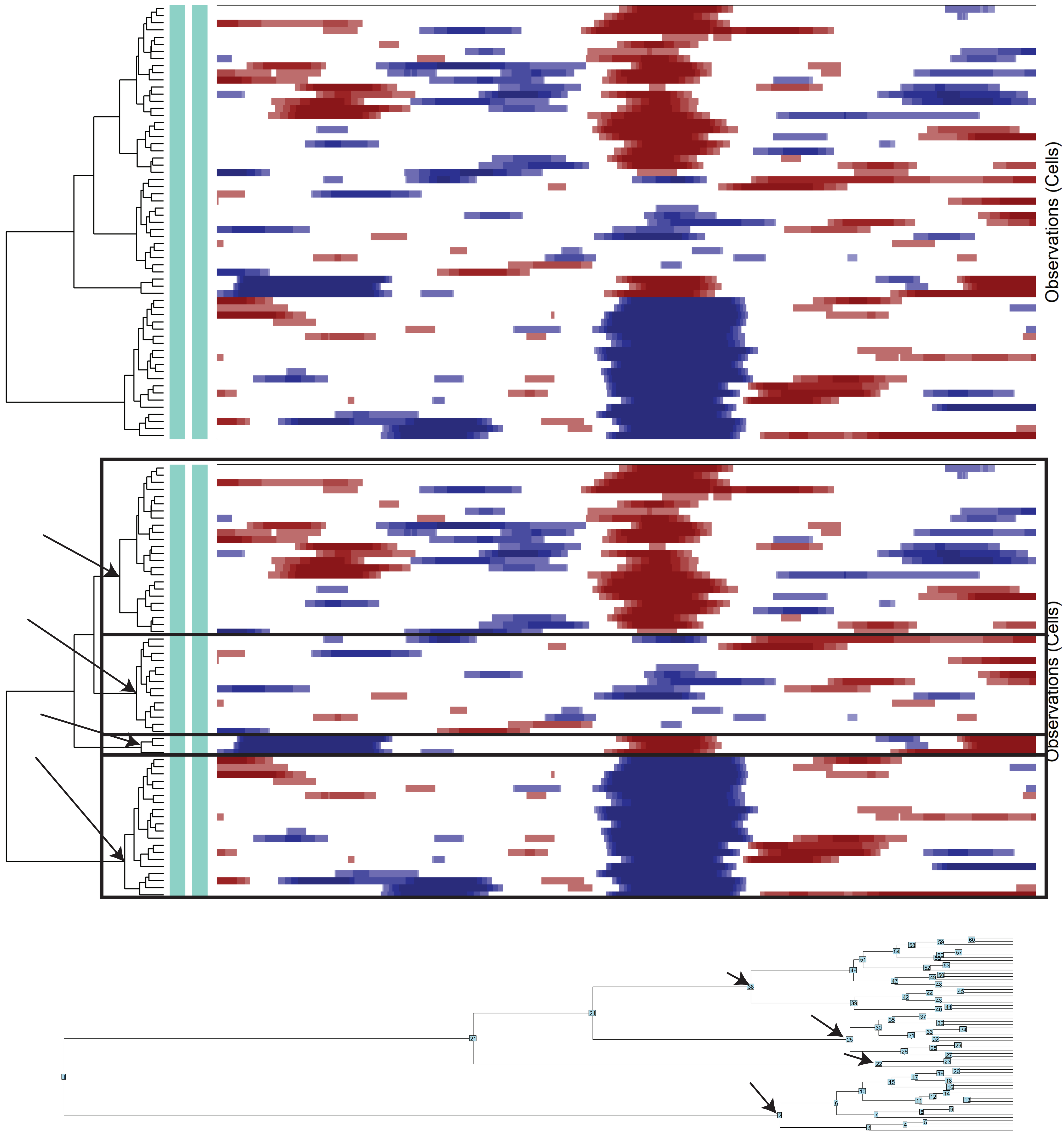

17_HMM_preds

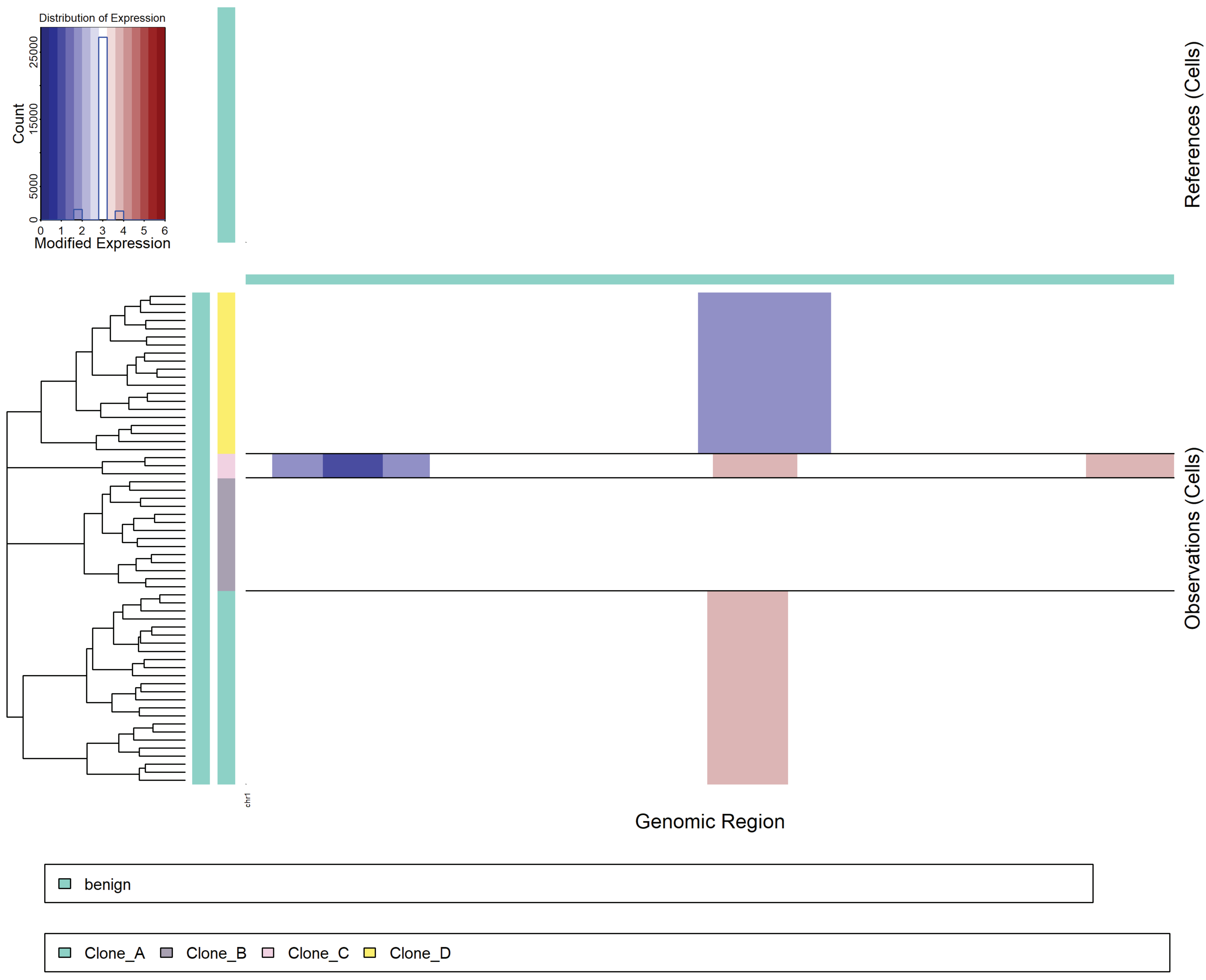

21_denoised

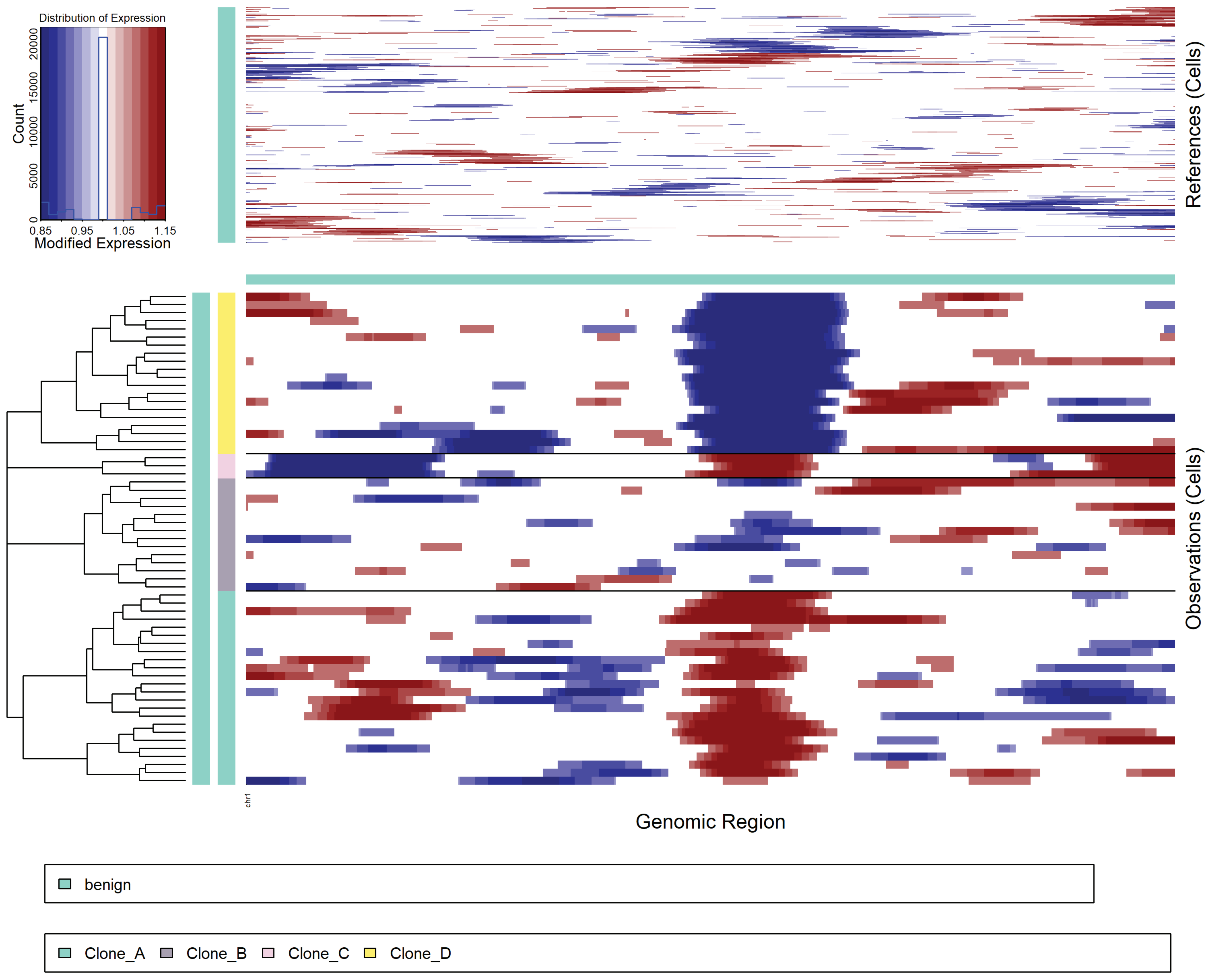

Supplement: Supplementary file 1 — Synthetic data generation and evaluation. Files related to method evaluation with synthetic data, such as the design file, the generated synthetic data, results from spatial inferCNV when applied to the synthetic data and a script for evaluation of results. [file CloneSelection_21052021.pdf › supplementary-data-S2/results/CloneSelection_21052021.pdf]
