# Supplementary material for: Spatially resolved clonal copy number alterations in benign and malignant tissue
Source: Nature. 2022 Aug 10;608(7922):360–7. doi: 10.1038/s41586-022-05023-2 (PMC9365699; doi:10.1038/s41586-022-05023-2)
Supplement: Supplementary file 2 — Reporting Summary [file 41586_2022_5023_MOESM2_ESM.pdf]

nature portfolio

Corresponding author(s): Joakim Lundeberg & Alastair Lamb

Last updated by author(s): Apr 28, 2022

Reporting Summary

Nature Portfolio wishes to improve the reproducibility of the work that we publish. This form provides structure for consistency and transparency in reporting. For further information on Nature Portfolio policies, see our Editorial Policies and the Editorial Policy Checklist.

Statistics

For all statistical analyses, confirm that the following items are present in the figure legend, table legend, main text, or Methods section.

n/a	Confirmed	
☐	☒	The exact sample size (*n*) for each experimental group/condition, given as a discrete number and unit of measurement
☐	☒	A statement on whether measurements were taken from distinct samples or whether the same sample was measured repeatedly
☐	☒	The statistical test(s) used AND whether they are one- or two-sided<br>*Only common tests should be described solely by name; describe more complex techniques in the Methods section.*
☐	☒	A description of all covariates tested
☐	☒	A description of any assumptions or corrections, such as tests of normality and adjustment for multiple comparisons
☐	☒	A full description of the statistical parameters including central tendency (e.g. means) or other basic estimates (e.g. regression coefficient) AND variation (e.g. standard deviation) or associated estimates of uncertainty (e.g. confidence intervals)
☐	☒	For null hypothesis testing, the test statistic (e.g. *F*, *t*, *r*) with confidence intervals, effect sizes, degrees of freedom and *P* value noted<br>*Give P values as exact values whenever suitable.*
☐	☒	For Bayesian analysis, information on the choice of priors and Markov chain Monte Carlo settings
☐	☒	For hierarchical and complex designs, identification of the appropriate level for tests and full reporting of outcomes
☒	☐	Estimates of effect sizes (e.g. Cohen's *d*, Pearson's *r*), indicating how they were calculated

Our web collection on statistics for biologists contains articles on many of the points above.

Software and code

Policy information about availability of computer code

Data collection	Histology images were captured using the Metafer Slide Scanning Platform (Metasystems). Raw images were stitched together with the VSlide Software (Metasystems).<br><br>Sequencing of spatial transcriptomics libraries were performed on illumina instruments using their proprietary platform and demultiplexed using DRAGEN.

Data analysis

The manuscript used publicly available, open source R and Python libraries/packages as described in the methods text. Two new libraries were developed for this manuscript, and are made available via Github (https://github.com/aerickso/SpatialInferCNV and https://github.com/almaan/growmeatissue).

Software and package version used during analysis:
Python (3.6.0)
R (4.1.3) with packages:
- Seurat (3.2.2)
- STUtility (0.1.0)
- SCTransform (0.3.3)
- tidyverse (1.3.1)
- infercnv (1.10.0)
- hdf5r (1.3.5)
- phangorn (2.8.1)
- Dendextend (1.15.2)
- msigdbr (7.4.1)
- fgsea (1.16.0)

For manuscripts utilizing custom algorithms or software that are central to the research but not yet described in published literature, software must be made available to editors and reviewers. We strongly encourage code deposition in a community repository (e.g. GitHub). See the Nature Portfolio guidelines for submitting code & software for further information.

Data

Policy information about availability of data

All manuscripts must include a data availability statement. This statement should provide the following information, where applicable:

- Accession codes, unique identifiers, or web links for publicly available datasets
- A description of any restrictions on data availability
- For clinical datasets or third party data, please ensure that the statement adheres to our policy

Count matrices, high-resolution histological images and additional material, are available on Mendeley Data (https://doi.org/10.17632/svw96g68dv.1).

Raw fastq for the prostate samples are available on request and is deposited to European Genome-Phenome Archive (EGA, www.ebi.ac.uk/ega/), which is hosted by the European Bioinformatics Institute (EBI) under the study: ID EGAS00001006124. The data are available under Data Use Conditions (DUO) and are limited to non-for-profit use as well as health/medical/biomedical purposes. Access is granted if the above is fulfilled and local institutional review board/ethical review board approvals are provided.

Raw fastq files for the childhood brain tumour samples are available through a Materials Transfer Agreement with Monica Nister (monica.nister@ki.se), in line with GDPR regulations.

Public data used for comparison of phylograms were obtained from European Nucleotide Archive (ENA; http://www.ebi.ac.uk/ena), accession numbers ERP022266 (RNA-seq) and ERP022267 (WGS) as well as from European Genome-phenome Archive (EGA; https://www.ebi.ac.uk/ega/), accession number EGAS00001001659 and EGAS00001000942. Public patient-specific benign cutaneous scRNAseq data were obtained from GEO (GSE144236). Public spatial transcriptomics data used in the study were all obtained from 10x genomics. Human lymph node (https://www.10xgenomics.com/resources/datasets/human-lymph-node-1-standard-1-1-0), breast cancer (https://www.10xgenomics.com/resources/datasets/human-breast-cancer-block-a-section-1-1-standard-1-1-0) and glioblastoma (https://www.10xgenomics.com/resources/datasets/human-glioblastoma-whole-transcriptome-analysis-1-standard-1-2-0) are all available as dataset resources.

Field-specific reporting

Please select the one below that is the best fit for your research. If you are not sure, read the appropriate sections before making your selection.

☒ Life sciences ☐ Behavioural & social sciences ☐ Ecological, evolutionary & environmental sciences

For a reference copy of the document with all sections, see nature.com/documents/nr-reporting-summary-flat.pdf

Life sciences study design

All studies must disclose on these points even when the disclosure is negative.

Sample size

This was a biological study and not a clinical trial and therefore we did not undertake a power calculation for the number of patients. For prostate, sample size of two was used as this is what was provided by the urologist. As a exploratory study of prostate cancer heterogeneity and showcase of spatial inferCNV this sample size was deemed sufficient.

Data exclusions

All patients analysed were included in the data presented. There were no excluded subjects.

Replication

All spatial transcriptomics experiments, including histology, of prostate samples were performed in technical replicates of two and a biological replicate in the form of an additional whole prostate. All samples and analyses confirmed the original findings. In addition, technical repeats of data analyses (spatial inferred CNV) was also re-run to confirm analysis results. smFISH and spatial transcriptomics experiments on other tissues were not repeated.

Randomization | This was a biological study and not a clinical trial and therefore we did randomize.

Blinding | This was a biological study and not a clinical trial therefore we did not blind. All samples processed contained prior known cancer tumors.

Reporting for specific materials, systems and methods

We require information from authors about some types of materials, experimental systems and methods used in many studies. Here, indicate whether each material, system or method listed is relevant to your study. If you are not sure if a list item applies to your research, read the appropriate section before selecting a response.

Materials & experimental systems

n/a	Involved in the study
☒	☐ Antibodies
☒	☐ Eukaryotic cell lines
☒	☐ Palaeontology and archaeology
☒	☐ Animals and other organisms
☐	☒ Human research participants
☒	☐ Clinical data
☒	☐ Dual use research of concern

Methods

n/a	Involved in the study
☒	☐ ChIP-seq
☒	☐ Flow cytometry
☒	☐ MRI-based neuroimaging

Human research participants

Policy information about studies involving human research participants

Population characteristics | Prostate patient 1 and 2 were both male at the age of 82 and 63 years old respectively. Both were diagnosed with prostate cancer and had radical prostatectomy performed. No genotyping was performed on the patients.

Recruitment | Candidate subjects diagnosed with primary prostate cancer whom were to undergo radical prostatectomy were identified and randomly selected by one of the study pathologists (AT). The two human subjects were provided with full and adequate verbal and written information about the study before their participation. Written informed consent was obtained from all participating subjects before enrolment in the study.

Ethics oversight | The study was performed according to the Declaration of Helsinki, Basel Declaration and Good Clinical Practice. The study was approved by the Regional Ethical Review Board (REPN) Uppsala, Sweden before study initiation (Dnr 2011/066/2, Landstinget Västmanland, Sari Stenius), Regional Ethical Review Board (EPN), Stockholm, Sweden (DNR 2018/3-31, Monica Nister).

Note that full information on the approval of the study protocol must also be provided in the manuscript.
